# A culturally adapted lifestyle intervention addressing a Middle Eastern immigrant population at risk of diabetes, the MEDIM (impact of Migration and Ethnicity on Diabetes In Malmö): study protocol for a randomized controlled trial

**DOI:** 10.1186/1745-6215-14-279

**Published:** 2013-09-03

**Authors:** Sanjib Saha, Matti Leijon, Ulf Gerdtham, Kristina Sundquist, Jan Sundquist, Daniel Arvidsson, Louise Bennet

**Affiliations:** 1 Health Economics and Management, Institute of Economic Research, Lund University, Lund, Sweden; 2 Department of Clinical Sciences, Center for Primary Health Care Research, Lund University/Region Skåne, Skåne University Hospital, Building 60, floor 12 Jan Waldenströms gata 37, 205 02 Malmö, Sweden; 3 Department of Economics, Lund University, Lund, Sweden; 4 Stanford Prevention Research Center, Stanford University School of Medicine, Palo Alto, CA, USA

## Abstract

**Background:**

Studies have shown that lifestyle interventions are effective in preventing or delaying the onset of type 2 diabetes in high-risk patients. However, research on the effectiveness of lifestyle interventions in high-risk immigrant populations with different cultural and socioeconomic backgrounds is scarce. The aim was to design a culturally adapted lifestyle intervention for an immigrant population and to evaluate its effectiveness and cost-effectiveness.

**Methods/design:**

In this randomized controlled trial, 308 participants (born in Iraq, living in Malmö, Sweden and at high risk of type 2 diabetes) will be allocated to either a culturally adapted intervention or a control group. The intervention will consist of 10 group counseling sessions focusing on diet, physical activity and behavioral change over 6 months, and the offer of exercise sessions. Cultural adaptation includes gender-specific exercise sessions, and counseling by a health coach community member. The control group will receive the information about healthy lifestyle habits provided by the primary health care center. The primary outcome is change in fasting glucose level. Secondary outcomes are changes in body mass index, insulin sensitivity, physical activity, food habits and health-related quality of life. Measurements will be taken at baseline, after 3 and 6 months. Data will be analyzed by the intention-to-treat approach. The cost-effectiveness during the trial period and over the longer term will be assessed by simulation modeling from patient, health care and societal perspectives.

**Discussion:**

This study will provide a basis to measure the effectiveness of a lifestyle intervention designed for immigrants from the Middle East in terms of improvement in glucose metabolism, and will also assess its cost-effectiveness. Results from this trial may help health care providers and policy makers to adapt and implement lifestyle interventions suitable for this population group that can be conducted in the community.

**Trial registration:**

ClinicalTrials.gov, NCT01420198

## Background

Type 2 diabetes (T2D), impaired fasting glucose (IFG), impaired glucose tolerance (IGT) and impaired glucose regulation (IGR) are among the strongest risk factors for cardiovascular disease (CVD) and death [1,2]. The prevalence of T2D is increasing, and it is estimated that by 2025, 15% of the global population will be affected by T2D, IFG or IGT [3]. The increase is thought to be due to population growth, ageing, urbanization, physical inactivity and obesity [4].

Immigrants from the Middle East and their offspring living in Sweden are at particularly high risk of T2D [5,6]. Malmö is a multicultural Swedish city. 30% of its residents were born abroad and the largest immigrant group consists of individuals born in Iraq, who collectively comprise almost 9,000 of Malmö’s nearly 300,000 inhabitants. In 2010, a pilot survey of almost 200 residents of Rosengård in Malmö who were born in Iraq or Sweden revealed a very high prevalence of T2D (20%), independent of country of birth, perhaps as a result of the study having been conducted in a socioeconomically vulnerable area [7]. Since January 2011, the MEDIM (the impact of Migration and Ethnicity on Diabetes In Malmö) project has continuously recruited residents with Iraqi and Swedish backgrounds aged 30 to 75 years from the whole of Malmö. Preliminary results obtained by June 2012 show that the prevalence of T2D is twice as high in Iraqi immigrants (n = 898) compared to Swedish participants (n = 757) (12.4% vs. 5.9%, p < 0.001), and that risk factors for T2D, such as family history of diabetes (52%), physical inactivity (64%) and obesity (38%), cluster in Iraqi subjects (manuscript submitted November 2012).

A previous study indicated that patients with T2D from the Middle East have a different form of T2D, with earlier onset and stronger family history function [8]. Furthermore, the same study indicated that immigrants from the Middle East have a more rapid decline in pancreatic beta-cell function compared to Swedish diabetic patients [8]. It is known that physical exercise enhance beta-cell function in people with diabetes [9] and also in people at high risk of diabetes or prediabetes [10]. However, little is known about how increased physical activity and improved lifestyle habits affect beta-cell function and/or insulin sensitivity in immigrants from Iraq.

These previous findings indicate that a large proportion of the Iraqi population is at risk of developing T2D within the next years. The high prevalence rates of family history of diabetes, obesity and sedentary lifestyle raise the need for a lifestyle intervention targeting individuals at risk of T2D. It is well established that diet- and physical activity-based lifestyle interventions are effective in preventing or delaying the onset of T2D in the short term [11,12] and in the long term [13-16]. However, there are currently no routines or programs to assist health care providers in improving lifestyle in individuals at risk of T2D who come from the Middle East and have a different cultural and socioeconomic background.

Recent studies highlight several obstacles and issues that need to be addressed in order to achieve successful lifestyle change in immigrants from the Middle East. Gender-specific groups must be offered [17]; physical activity sessions should be adapted to the preferences of the participants [18]; successful intervention models should be provided by a multidisciplinary team [18]; and the intervention models should mediate behavioral change and self-empowerment [18]. Therefore, there is a need for a culturally adapted lifestyle intervention targeted to Iraqi immigrants that might differ from conventional lifestyle interventions.

In addition to the challenge of designing a culturally adapted lifestyle intervention, a further challenge remains in the implementation of the lifestyle intervention in community and primary care settings. Every intervention costs resources, but resources are always scarce and informed decision-making is required before implementation of a program at the mass population level. Cost-effectiveness analysis can ensure that limited resources are allocated as efficiently as possible, so that decision makers can take informed decisions based on the benefits outweighing the costs [19]. Although active screening for T2D combined with subsequent lifestyle intervention is cost-effective [20] compared to the “wait and see” or “do nothing” approach that currently prevails, no study has emphasized the cost-effectiveness of culturally adapted lifestyle interventions for high-risk immigrants from Iraq.

## Aim/purpose

Immigrants to Sweden from Iraq are at high risk of developing T2D, but little is known about factors preventing the development of T2D in this group. The aims of the study are to:

(1) Develop a culturally and primary health care-adapted lifestyle intervention program focusing on increased levels of physical activity and healthier eating habits.

(2) Study if the program has an effect on glucose metabolism (measured as fasting glucose level) and whether the change in fasting glucose is depending on insulin sensitivity index (ISI), insulin secretion (measured as oral disposition index (DIo)) and/or family history of diabetes.

(3) Explore factors that can predict beneficial and non-beneficial responses to the lifestyle intervention, such as baseline physical activity, motivational level, attitudes towards lifestyle change and adherence to intervention components.

(4) Study the cost-effectiveness of this lifestyle intervention from short term and long-term time horizon with patient, health care and societal perspectives.

## Methods/design

### Study design

The study is a 6-month randomized controlled lifestyle behavior intervention trial, where intervention and control groups are followed at 3 and 6 months. Written informed consent will be obtained before randomization. Eligible participants not willing to participate will be registered as such.

### Participants

#### Eligibility criteria

Participants are recruited from the MEDIM baseline study that by December 2012 had included 1398 men and women aged 30 to 75 years born in Iraq, and in whom a glucose tolerance test was performed and sociodemography and lifestyle was characterized. In total 40% of the participants are estimated to be at risk of diabetes either by having prediabetes (IFG, IGT or IGR) or by their constellation of risk factors according to FINDRISC scores [21] resulting in a moderate (17%) to high risk (33%) to develop diabetes within the next decade. Thus we will have an eligible population of 1398*0.40 = 560 individuals, of whom 60% or 336 are estimated to finally agree to participate in the study. The participation rate is based on experiences from an intervention study of Arabic women, which had a participation rate of 62% [17]. In the baseline study 315 Iraqi individuals had a moderate risk (i.e. an estimated 10 year incidence rate of diabetes of 17%) [21] and 275 individuals had a high risk (i.e. an estimated 10 year incidence rate of diabetes of 33%) [21]. Based on a mean time of two years from baseline to the start of the study, we estimate that 26 individuals may have developed diabetes during this period. They will consequently be excluded from the study. Based on a drop-out rate of about 16%, we estimate that of the 310 individuals that initially participated 260 will complete the study (130 per group).

Inclusion criteria were being born in Iraq, age 30 to 75 years and high risk of diabetes as measured by

A. IFG, IGT or IGR identified by an oral glucose tolerance, plasma glucose (glc) at fasting (f-glc) and at 2 hours (2-h glc) during glucose tolerance test:

•  IFG: f-glc 6.1-6.9 mmol/L (110.125 mg/dL) and 2-h glc <7.8 mmol/L (<140 mg/dL)

•  IGT: f-glc < 6.1 mmol/L (<110 mg/dL) and 2-h glc 7.8-11.0 mmol/L (140.199 mg/dL)

•  IGR: f-glc . 6.9 mmol/L (.125 mg/dL) and 2-h glc 7.8-11.0 mmol/L (140.199 mg/dL)

B. or by Finish diabetes risc score (FINDRISC) with over 10% risk of diabetes in 10 years.

Exclusion criteria were diabetes, current pregnancy, mental incapability and/or cognitive impairment, current CVD or history of CVD events. CVD includes stroke, angina or myocardial infarction (MI), percutaneous transluminal coronary angioplasty (PTCA), congestive heart failure (CHF), coronary artery bypass graft surgery (CABG), transient ischemic attack (TIA) and peripheral vascular disease (PVD) or other physical disorders that prevent physical exercise.

#### Randomization/blinding

Subjects agreeing to participate will be randomly allocated to the intervention (n = 155) or control group (n = 155). Randomization will be stratified by age, gender, prediabetes and FINDRISC score, with the aim of obtaining an equal distribution of participants between the two groups. Spouses will be randomized to the same group, and spouses not fulfilling the inclusion criteria will be invited to participate in the study by supporting their husband or wife.

#### Intervention group

The intervention group will be offered (A) regular group sessions led by an Arabic-speaking health coach with a similar ethnic background to the group and (B) free physical activity sessions. Others showed that the willingness to enroll in a diabetes prevention program was predicted by perceived risk of diabetes [22] and before the start of the study we will hold an education session to provided information about how our lifestyle habits influence our health.

#### Control group

Information will be sent to the primary health care center (PHCC) that the participant is at high risk of T2D and that he/she will participate in the study as control. The control group will receive “treatment as usual”, *i.e.* they will be instructed by nurses in the PHCC to increase their physical activity, to reduce their weight and to make the same diet changes as those recommended to the intervention group.

#### Intervention

**Figure 1:**
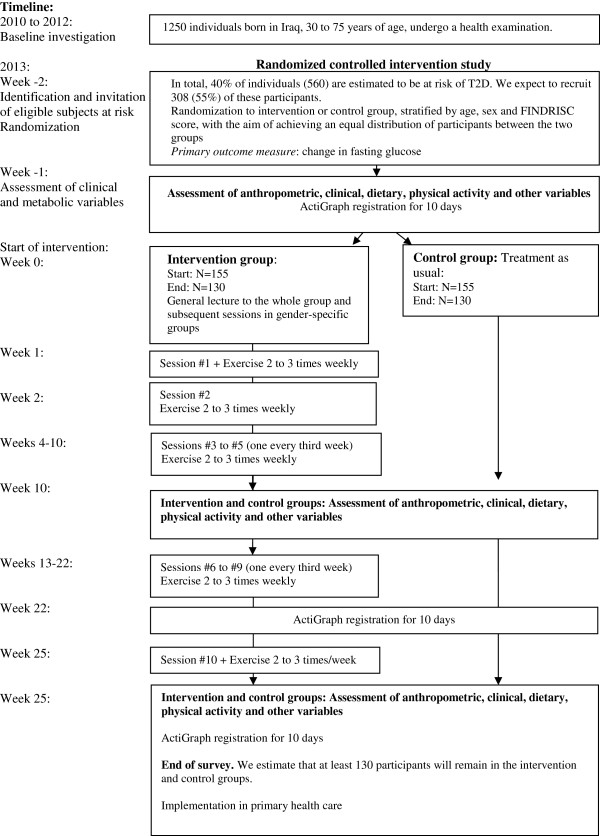
Flow chart summary of the 6-month lifestyle intervention study.

The intervention will be based on evidence-based guidelines for the prevention of T2D [23]. These guidelines include a combination of physical activity and dietary intervention and focus on behavior maintenance. Physical activity and diet counseling will be conducted in gender-specific groups by a multidisciplinary team. In order to be easily implemented, the program will be adapted to present resources in primary health care and the community. Figure 1 shows a flow chart summary of the study.

Ten group counseling sessions will be provided during a period of 6 months. Each group will contain 16 individuals of the same gender (there will be five groups of men and five groups of women). The sessions target diet, physical activity and behavioral change with the goal of equipping the subjects with the necessary knowledge and skills to gradually achieve permanent behavioral changes. Behavioral change is a model involving *motivation*, *action* and *maintenance*[24]. It supports changes in diet and physical activity and goal-setting, and involves action planning with goals and coping strategies. The intervention group will be supported in their efforts to reach the following goals, which are considered to have preventive effects against diabetes [15,24]: weight reduction of ≥5%, moderate intensity physical activity ≥30 minutes at least 5 days/week, dietary fat <30% of total energy (E%), saturated fat <10 E% and fiber ≥15 g/1000 kcal. The participants will also be encouraged in everyday life to reduce their time spent physically inactive, *e.g.* sitting or lying down, as much as possible. The intervention group will be encouraged to regularly fill in a “smart goal” sheet to help them to specify their goals (such as to lose 0.5 kg per week), and to explain why they want to do it (for example “*I don’t want to get diabetes*”), how they will reach their goals (“*I will walk briskly for 30 minutes every day after work*”) and the coping plan they will apply to conquer different possible obstacles that may appear (“*I will ask my grandmother to come over and babysit while I am away at practice*”), as suggested in the European guidelines to prevent diabetes [24].

#### Group counseling sessions

The participants will have group consultations with the Arabic speaking health coach and/or a nutritionist on preplanned topics, such as how lifestyle affects diabetes and cardiovascular risk, healthy lifestyle habits, goal setting and problem solving, with open discussions focusing on individual problems and behavioral change. In addition, there will be between-visits phone calls to encourage and motivate the participants to maintain their new lifestyle habits.

#### Exercise sessions

Previous studies have shown beneficial effects of aerobic and resistance training on glucose metabolism in subjects with IGT [25,26]. In this study, physical activity will be prescribed using physical activity on prescription (in Swedish “*FaR*” [27]. The participants will be encouraged to participate in a physical activity program combining resistance exercise and endurance training two to three times a week to increase their possibility of reaching outcome goals. The resistance exercise training will be performed with their own body weight, rubber bands and dumbbells and aims to increase muscle strength and endurance. The aerobic endurance training will comprise self-selected activities, such as aerobic classes, swimming, soccer, jogging or walking led by certified instructors. As far as possible, the endurance classes and resistance training classes will be supervised by a leader from Iraq of the same gender as the group. Participation lists will be filled in by the supervisors of the physical activity classes.

#### Assessment and outcome measures

Trained Swedish- and Arabic-speaking research nurses will conduct the measurement procedures. Measurement will be conducted at baseline, in the middle of intervention (3 months) and at the end of intervention (6 months). Table 1 shows a schedule for the assessments that the participants will undergo during the study period.

**Table 1:** Schedule of assessments and assessment measures

	**Screening**	**Baseline**	**Months**	
			**3**	**6**
**Anthropometrical**				
Height	x	x		
Weight	x	x	x	x
Waist circumference	x	x	x	x
Buttock circumference	x	x	x	x
**Clinical**				
Fasting plasma glucose	x	x	x	x
Cholesterol	x	x	x	x
Triglycerides	x	x	x	x
HDL-C	x	x	x	x
LDL-C	x	x	x	x
HbA1c	x	x	x	x
C-peptide	x	x	x	x
s-insulin	x	x	x	x
OGTT	x	x	x	x
**Physical cctivity**				
IPAQ		x	x	x
ActiGraph	x	x	x	x
VO_2_ max		x	x	x
**Dietary**				
Food record		x	x	x
**Physical and mental wellbeing**				
EQ-5D		x	x	x
Hospital Anxiety and Depression Scale	x	x	x	x
(HADS)				
**Questionnaire**				
Sociodemographics	x	x		
Stage of change		x	x	x
Self-determination theory (TSRQ)		x	x	x
Alcohol habits (AUDIT)	x	x	x	x
Tobacco habits	x	x	x	x
Cost				x
Willingness to pay (WTP)				x

#### Sociodemographic measurements

Sociodemographic data will be collected at baseline only and will include age, education level, household income, employment status, marital status, years spent in Sweden, language spoken at home and with friends, complete family history of diabetes in first- and second-degree relatives, current and previous illness and co-morbidities, and current medications.

#### Anthropometrical measurements

Height will be measured to the nearest centimetre using a wall-mounted stadiometer. Weight will be measured to the nearest kilogram with participants wearing light clothing, but not shoes, using a calibrated 34–5062 RTC3010 electronic scale (Coline, China). Body mass index (BMI) will be calculated as weight (kg) divided by height squared (m^2^). Waist circumference will be measured twice with a tape to the nearest 0.1 cm at the midpoint between the lowest rib margin and the iliac crest. Buttock (hip) circumference will be measured where the protrusion of the buttocks is greatest.

#### Biomedical measurement

Participants will be instructed not to eat or drink anything but water or consume tobacco after 10 pm the day before testing and to bring a record of their current medications. Blood pressure will be measured in the supine position after five minutes’ rest and with the arm at heart level. The mean of two measurements, taken one minute apart, will be used in analyses. Blood samples will be collected in the morning and analyzed continuously during the study. Serum insulin (s-insulin) levels will be measured by Access^©^ Ultrasensitive Insulin radioimmunoassay (Beckman Coulter, USA) [28]; serum cholesterol and triglyceride levels will be measured using enzymatic methods (Bayer Diagnostics) [29]. Serum HDL-cholesterol levels will be measured enzymatically after isolation of LDL and VLDL (Boehringer Mannheim GmbH, Germany); LDL-cholesterol levels will be estimated using Friedewald’s equation [30]. C-peptide levels will be measured with a one-step immunometric sandwich electrochemiluminescence immunoassay (ECLI) based on a ruthenium (Ru) derivative (Roche). A standard 75-g OGTT will be performed and blood samples will be collected at 0, 30, 60 and 120 min. Blood glucose (f-glucose) will be measured in venous whole blood immediately after sampling using a photometer (HemoCue AB, Ängelholm, Sweden) [31]. HbA1c will be estimated by high-pressure liquid chromatography (HPLC) with a VARIANT™ TURBO Hemoglobin A1c Kit 2.0 (Bio-Rad). ISI, corrected insulin response (CIR) and DIo will be calculated from the OGTT results. ISI will be calculated as follows:

$$\begin{array}{ll} \mathrm{ISI}= & 10,000/\surd [\left(f-\mathrm{glucose}\;\left(\mathrm{mmol}/L\right)\times s-\mathrm{insulin}\;\left(\mathrm{mIE}/L\right)\right)\;\\ & \times \left(\mathrm{mean}\;\mathrm{OGTT}\;\mathrm{glucose}\;\mathrm{conc}.\left(\mathrm{mmol}/L\right)\right)\\ & \left(\times \mathrm{mean}\;\mathrm{OGTT}\;\mathrm{insulin}\;\mathrm{conc}.\left(\mathrm{mIE}/L\right)\right)]\; \end{array}$$

[32].

CIR provides an estimation of beta-cell function and will be calculated as follows:

$$\mathrm{CIR}=(100\times \mathrm{insulin}\;\mathrm{at}\;30\;\min/\left(\mathrm{gluc}30\times \left(\mathrm{gluc}30–3.89\right)\right)$$

[33], where gluc30 is glucose at 30 min and must be >4.44 mmol/l and >f-glc [34].

DIo provides an estimate of beta-cell function adjusted for insulin resistance, and is calculated as CIR multiplied by ISI [35].

#### Physical activity and cardiorespiratory fitness

We will use two types of physical activity measurement methods: self-report and objective. The self-administered, long version of the International Physical activity Questionnaire (IPAQ) will be used to assess self-reported physical activity level, as it may provide the most reliable data for the present study [36]. IPAQ is one of the most used and evaluated questionnaires [36]. However, self-report methods may be subject to recall bias and may provide different results compared to objective instruments such as accelerometers [37]. Therefore, we will also use ActiGraph accelerometers, which are most commonly used in research to provide an objective measure of duration, intensity and frequency of physical activity. Physical activity during all waking hours in a 10-day period will be measured every second month with ActiGraph GT3X accelerometers (Pensacola, Florida, USA). The GT3X accelerometer provides sufficient storage capacity for the measurement period, shows high intra- and inter-monitor reliability [38,39] and may capture a wide range of physical activity intensities [40,41]. A period of 10 days provides sufficient information to capture the variation in physical activity [42].

Cardiorespiratory fitness will be assessed by measuring maximum aerobic capacity (VO_2_ max). Subjects will undergo a 6-minute walking test that provides an indirect estimate of oxygen uptake [43] and that has been validated in subjects up to 65 years of age [44]. Performance is estimated by measuring heart rate and walking time at the end of the test, with adjustment for age, gender and BMI. A prerequisite for conducting the test is the ability to walk briskly and to achieve a heart rate of ≥70% of the age-related maximal heart rate.

#### Dietary measurements

The participants will fill in forms with questions covering frequency and estimated level of intake of different foods (such as meat, fish, vegetables, potatoes, rice and cereals fruit, and different snacks), and how the food was prepared (cooked, deep fried, fried), to capture total food intake and habits (breakfast, lunch, dinner, snacks and beverages) during the last three days. Since there are no validated food questionnaires for Arabic foods and food habits, the questionnaires will be developed in collaboration with nutritionists and Arabic-speaking health coaches with knowledge about Arabic food habits and culture. The amount of food will be estimated by the participants by using “*Måltidsmått*” from the pharmacies in Sweden (a picture of *Måltidsmåttet* is presented at http://www.maltidsmattet.se/), which provides a measure of the volume of food. Special attention will be given to food habits during weekends and holidays. The food questions will be analyzed for nutrient content using Dietist XP software. The software contains food tables from the Swedish National Food Administration and includes almost 1600 food items and 50 nutrients [45].

#### Tobacco use

Daily smoking increases the risk of 58 different conditions (including cancer, CVD and pulmonary disorders), which together account for almost 10% of the total disease burden in Sweden [46]. Current smoking status (smoker or non-smoker) will be assessed, together with number of cigarettes per day for smokers. In addition, participants will be asked whether they use snuff or smoke water pipes.

#### Alcohol use

The self-reported version of the Alcohol Use Disorders Identification Test (AUDIT) from the World Health Organization (WHO) [47] will be used to assess alcohol intake habits. A total score of ≥8 will be used as an indicator of hazardous and harmful alcohol use, as well as possible alcohol dependence.

### Physical and mental well being

#### Health-related quality of life

Health-related quality of life will be measured using the EQ-5D questionnaire [48]. All participants (intervention and control groups) will complete the Arabic version of the questionnaire at the start, middle and at the end of the study.

#### Hospital anxiety and depression scale

We will use the Hospital Anxiety and Depression Scale (HADS) [49] to measure the level of anxiety and depression that each participant is experiencing before implementation of the lifestyle intervention. Although HADS was initially developed for hospital patients, it is valid for primary health care patients [50], as is the Arabic version as well [51] that will be used in this study.

### Motivation and behavioral change

#### Stage of change questionnaire

Stage of change is a model developed by Prochaska and colleagues [52]. It identifies five stages that individuals cycle through as they change specific “health-risk” behaviors and the individuals move through a series of stages in their attempts to accept the desired behavior. The stages represent a period of time as well as a set of tasks needed for movement to the next stage. The stage of change questionnaire will be used to identify each participants stage in their change towards healthy lifestyle habits (Table 2) and special care will be provided to treat them accordingly [53]. The scientific evidence for the effect of stage-targeted interventions for individual factors (*e.g.* smoking cessation, fat intake reduction) is strong, but the evidence for complex lifestyle interventions is limited [54].

**Table 2:** Stage of change questionnaire

**Response**	**Stage**
I am currently not very physically active and I do not intend to become more physically active during the next 6 months	Precontemplation
I am currently not very physically active, but I’ve been thinking about increasing my activity level during the next 6 months	Contemplation
I am currently not very physically active, but I am determined to increase my activity level during the next 6 months	Preparation
I am currently physically active, but I’ve only been so for the last 6 months	Action
I am currently physically active and I have been so for longer than 6 months	Maintenance
I was physically active a year ago, but in recent months I have been less active	Relapse
I do not know	

#### Self-determination theory

Self-determination theory (SDT) is a general theory of human motivation that aims to explain individuals’ goal-directed behavior. It explains the extent to which behaviors are autonomous (*i.e.* originate from the self) and the extent to which they are controlled by societal pressure and intrapsychic or interpersonal forces [55]. There is consistent evidence of the value of using SDT to understand physical activity behavior [56]. For the current study, we will use the treatment self-regulation questionnaire (TSRQ), which was designed to assess the different forms of motivation within SDT to determine participants’ motivation to adopt healthy lifestyle habits. The TSRQ has been validated for physical activity and healthy eating habits [57].

### Costs

Direct medical costs, direct non-medical costs and indirect costs will be calculated for both the intervention and control groups. Direct medical costs represent expenditures for medical services and products and are usually paid for by the health systems. These costs include the cost of hospitalization, outpatient care, laboratory tests and medications. Direct non-medical costs include time costs of participation in the exercise program, shopping, food preparation and cooking, and costs of exercise equipment, special foods and transportation. Indirect costs are productivity loss costs due to diseases.

Costs will be further divided into those borne by the program and those incurred by the study participants. Program costs include training costs and costs associated with setting up and performing the intervention. They also include resources required for meetings, staff preparation, meeting time and materials for the participants. Overhead and administrative costs will also be calculated according to recommended methods. Intervention providers (health coaches, nutritionists, physiotherapists, nurses, medical doctors, etc.) will be interviewed to determine the time they spent on each participant and the cost will calculated from their salary chart sheet. For cost calculation, we will use previously developed and validated questionnaires of the Diabetes Prevention Program (DPP) [13]. Each participant will be asked about the specific costs of their transport to and from the health care center, time costs and informal care costs (costs incurred by accompanying persons). Each participant will be asked about their sickness leave in the past two months. Participants will report their general sickness absence in general and also sickness absence due to physical exercise-related injuries. Lost days of productivity will be converted into costs by the human capital method to get the indirect cost.

### Economic evaluation

Cost-effectiveness analysis will be performed from patient, health care and societal perspectives. In the societal perspective, all costs are included irrespective of who is burdened by them, while the health care perspective is only concerned with costs burdening the health care sector. The patient perspective focuses on the costs that the patients have to bear to change their lifestyle habits through the intervention. The societal perspective is generally preferred, although a health care perspective supplies additional (affordability) information and can strengthen conclusions based on the societal perspective. The results will be presented in terms of incremental cost-effectiveness ratios (ICERs), which show the change in costs for an incremental benefit [19]. To determine the cost-effectiveness of the intervention, ICERs will be calculated for all statistically significant outcomes. In cost utility analysis, utility values will be calculated using the EQ-5D questionnaire which provides the health related quality of life as quality adjusted life years (QALY).

We will perform economic evaluation in two different time horizons; short-term (within-trial) and long-term. Within-trial economic evaluation will only focus on the trial cost data and the outcomes. For long-term duration, we will develop a decision analytic model based on the outcome of the trail. This will be done by extrapolating the secondary clinical outcomes (BMI, blood pressure, lipid profile) to predict future disease events such as CVD, diabetes and diabetes-related complications. Each disease event will have a future cost and QALY. Future costs and effects will be discounted by 3% per year, but we intend to perform sensitivity analyses on other rates [58]. We will analyze uncertainty by both one-way and multi-way sensitivity analysis, and also by non-parametric bootstrapping. We will calculate 95% confidence intervals for the ICERs.

We will also calculate the willingness to pay (WTP) to be in the program of the participants by the contingent valuation method. In this method, participants are directly asked what they are willing to pay for a benefit or service [19]. There are many ways to formulate WTP *i.e.* open-ended, closed-ended or biding game type [59]. For the current study, we will use closed-ended questions with predetermined maximum WTP options. The monetary value will represent the annual subscription fee for several commercial gymnasiums in Sweden. Participants will be asked to give reasons for their willingness and unwillingness to pay. The methodology will follow that used in two previously published studies with similar lifestyle interventions [60,61].

### Process evaluation

To assess adherence to the intervention protocol, attendance at group counseling sessions and exercise sessions will be registered, as will accomplishment of individual activities (*e.g.* smart goal sheet, *FaR*).

### Cultural adaptation

Special emphasis will be placed on adapting the intervention to the cultural norms, beliefs and traditions of the participants from the beginning to the end of the study period. In order to achieve successful lifestyle change, gender-specific education exercise groups will be offered and physical activity sessions will be adapted to the preferences of the participants [17,18]. Moreover, the intervention will be provided by a multidisciplinary team comprising a health coach, nurses, a physiotherapist and a doctor, and mediates behavioral change and self-empowerment that has been shown to improve the response to lifestyle change in ethnic minority groups [18].

Study materials, including the informed consent form, participant information, food booklets, and newsletters, will be translated into Arabic. Questionnaires only available in English or Swedish will be translated into Arabic according to WHO guidelines for translating instruments [62] and will be administered by a health care professional who can speak both Swedish and Arabic.

## Statistical analysis

### Sample size and power calculation

#### Fasting glucose

From the baseline study and earlier intervention studies on Arabic participants, we estimate a standard deviation (SD) of fasting glucose of 0.6 [12,63]. Based on earlier studies, we assume that the true difference between the intervention and control group means is 0.22 mmol/L [12,63]. We will be able to reject the null hypothesis that the population means of the intervention and control groups are equal with a power of 0.86.

#### Cost-effectiveness analysis

It is assumed that the point estimates for differences in mean cost and QALY between the intervention and control groups will be 1000 SEK and 0.18 QALY, respectively, with differences in the SDs for cost and QALY of 5000 SEK and 0.26 QALY, respectively. The sample size of 260 (130 per group) provides 0.84 power, assuming that the WTP is 1.3 million SEK [64,65].

### Univariate and multivariate analysis

The trial data will be analyzed on an intention-to-treat basis. Bivariate and multivariate analyses will assess the effects of the intervention (compared to controls) on fasting glucose level, weight, physical activity level and dietary intakes, with adjustment for age and gender, and will identify factors that can predict beneficial and non-beneficial responses to the intervention (*e.g.* baseline physical activity, motivation, attitude, adherence to the intervention). Continuous data will be analyzed with paired Student’s t-tests and linear regression, and categorical variables with chi square tests and logistic regression with 95% confidence intervals.

### Follow up and evaluation of data

The intervention and control groups will be followed up every 3 months for BMI, fasting glucose, HbA1c, blood pressure, diet registration, physical activity and fitness, and with questionnaires. The final evaluation conducted after 6 months will also include an OGTT.

We will study whether the intervention has a significant effect on fasting glucose levels and whether this effect is depending on ISI, DIo or family history of diabetes. We will also study factors that may predict these changes, including intensity and amount of physical activity (measured with ActiGraph accelerometers and questionnaires) and behavioral factors such as stage of change and motivation level (measured with questions originating from self-determination theory).

### Ethical approval

The study protocol and informed consent form (in Arabic) were approved by the Ethical Review Board of Lund University, Sweden (approval no. 2011/88). The blood samples are stored in biobank BD35, the Clinical Research Centre, Region Skåne, Sweden. Access to the blood samples for other researchers will be regulated by ethical approvals.

## Discussion

This article presents a detailed description of a culturally adapted randomized controlled trial of a lifestyle intervention, whose effectiveness in combating risk factors for T2D and cost-effectiveness we will investigate in Iraqi immigrants.

Although several resources for lifestyle change are today available in primary health care in Sweden, these programs are not culturally adapted and individuals at high risk of T2D are consequently not given priority. A considerable proportion of Sweden’s inhabitants are immigrants from the Middle East, who have been identified as a group at high risk of T2D. Instead of just waiting for these high-risk individuals to develop T2D, the study seeks to optimize preventive action in the community and primary health care and aims to facilitate the adoption of changes in lifestyle in high-risk patients, taking account of cultural and social barriers. A community- and primary health care-oriented intervention model based on a successful evidence-based approach targeting individuals at high risk of T2D with a different cultural background will increase the probability of successful preventive actions in the community and primary health care in this high-risk group.

There are some potential challenges that may arise during this study. One challenge is to motivate Muslim women to participate in physical exercise as the baseline study have shown that 70% are physically inactive and may be reluctant to engage in physical exercise [66]. We aim to solve this issue by offering gender-specific group sessions with a health coach of the same gender as the participants. A limitation could be that the physical activity sessions are adapted to the preferences of the participants, which may be in conflict with the goals of achieving physical activity. Therefore, this study emphasizes increasing the motivational level of the participants by working on behavioral change.

The MEDIM intervention study will contribute with an intervention program that is adapted not only to evidence-based guidelines but also to Middle Eastern cultural and lifestyle habits, which is crucial for the program to succeed and reach its goals. The project has a strong clinical approach, focusing on clinical diabetes prevention research, and aims to provide a cost-effective method to decrease diabetes risk in a high-risk population. Increased knowledge in this novel research area would increase our understanding of the complex mechanisms that contribute to the high diabetes risk in Iraqi immigrants. The knowledge generated through this study will have important implications for health-related costs, quality of life and health equity.

## Trial status

This trial will recruit participants from October 2013.

## Competing interests

There authors declared that they have no competing interst.

## Authors’ contributions

SS wrote the manuscript and is the principal investigator for economic evaluation; ML participated in the study design and in writing the manuscript; UG, KS and JS contributed to the discussion, and reviewed and edited the article; DA participated in the study design, with a focus on physical activity, and reviewed and edited the article; LB is the principal investigator for the MEDIM study, conceived the study and its design, secured its funding, wrote the manuscript and is providing the leadership and coordination for the study. All authors have read and approved the final submitted manuscript.
